# Supplementary material for: Safety Profile of the 4CMenB (Bexsero®) Vaccine: A Systematic Review and Meta-Analysis of Adverse Events in Clinical Trials
Source: Vaccines (Basel). 2025 Oct 2;13(10):1030. doi: 10.3390/vaccines13101030 (PMC12568221; doi:10.3390/vaccines13101030)
Supplement: Supplementary file 1 [file vaccines-13-01030-s001.zip › vaccines-3888800-supplementary.pdf]

Table S1. List of studies excluded from the meta-analysis and reasons for exclusion

Study	*Reference*	*DOI*	*Design and Population*	*Reason for Exclusion*
1	Martinon-Torres F et al. Hum Vaccin Immunother. 2025;21(1):2463194.	10.1080/21645515.2025.2463194	Phase 2b RCT in healthy infants	Evaluated MenABCWY vaccine; did not analyze effects of 4CMenB independently.
8	Martinón-Torres F et al. J Infect. 2018;76(3):258-269.	10.1016/j.jinf.2017.12.005	Phase IIIb RCT in infants and previously vaccinated children	Did not report independent adverse effects of 4CMenB.
9	Martinón-Torres F et al. Vaccine. 2017;35(28):3548-3557.	10.1016/j.vaccine.2017.05.023	Phase 3b RCT in infants and children (26 centers)	Adverse effects are shown in figures without precise numerical data.
10	Safadi MAP et al. Vaccine. 2017;35(16):2052-2059.	10.1016/j.vaccine.2017.03.002	Phase 3b RCT in Brazilian infants	Evaluated 4CMenB and MenC-CRM jointly.
12	Findlow J et al. Vaccine. 2015;33(29):3322-30.	10.1016/j.vaccine.2015.05.027	Phase II RCT in adult laboratory workers	Adverse effects are presented in figures without detailed data.
14	Esposito S et al. Hum Vaccin Immunother. 2014;10:2005-14.	10.4161/hv.29218	Phase II RCT in infants	Data already included in study 15; incomplete information.
16	Vesikari T et al. Lancet. 2013;381(9869):825-35.	10.1016/S0140-6736(12)61961-8	Phase III RCT in infants (70 European centers)	No group with 4CMenB monotherapy.
17	Riedmann EM. Hum Vaccin Immunother. 2013;9(1):8-9.	10.4161/hv.23607	News about regulatory approval	Not a clinical trial with results.
18	Santolaya ME et al. Lancet. 2012;379(9816):617-24.	10.1016/S0140-6736(11)61713-3	Phase 2b/3 RCT in Chilean adolescents	Cumulative data without breakdown by adverse event.
20	Thornton V et al. Vaccine. 2006;24(9):1395-400.	10.1016/j.vaccine.2005.09.043	Phase I/II RCT in healthy adults	Evaluated a different vaccine than 4CMenB (MenBvac™).

Table S2. Frequency of local erythema as an adverse effect of the 4CMenB vaccine in clinical trials

Study Number	Author	Year	Number of cases	Total number	Frequency (%)	95% CI
2	Beran	2021	10	94	10,6	4 - 17
3 (Fase 1)	Vesikari	2021	23	227	10	6 - 14
3 (Fase 2)	Vesikari	2021	22	126	17	11 - 24
5	Nolon	2019	29	254	11	7 - 15
6	Macias	2018	102	228	45	38 - 51
7	Chiu	2018	54	148	36	29 - 44
11 (Lote 1)	Perrett	2015	81	169	48	40 - 55
11 (Lote 2)	Perrett	2015	75	173	43	36 - 51
13	McQuaid	2015	47	50	94	87 - 99
15	Prymula	2014	59	182	32	26 – 39
**Total**			502	1651	**30**	**28 – 33**
**Test of Homogeneity: P < 0.05 Significant**						

Table S3. Frequency of edema as an adverse effect of the 4CMenB vaccine in clinical trials

Study Number	Author	Year	Number of cases	Total number	Frequency (%)	95% CI
2	Beran	2021	12	94	12,8	6 – 20
5	Nolon	2019	43	254	17	12-21
6	Macias	2018	86	228	38	31-44
7	Chiu	2018	34	148	23	16-30
11 (Lote 1)	Perrett	2015	51	169	30	23-37
11 (Lote 2)	Perrett	2015	43	173	25	18-31
13	McQuaid	2015	19	50	38	25-51
15	Prymula	2014	32	182	18	12-23
Total			320	1298	**25**	**22 – 27**
**Test of Homogeneity: P < 0.05 Significant**						

Table S4. Frequency of local induration as an adverse effect of the 4CMenB vaccine in clinical trials

Study Number	Author	Year	Number of cases	Total number	Frequency (%)	95% CI
2	Beran	2021	12	94	12,8	6 – 20
3 (Fase 1)	Vesikari	2021	24	227	11	7-15
3 (Fase 2)	Vesikari	2021	20	126	16	9-22
5	Nolon	2019	43	254	17	12-21
6	Macias	2018	102	228	45	38-51
7	Chiu	2018	63	148	43	33-51
11 (Lote 1)	Perrett	2015	42	169	25	18-31
11 (Lote 2)	Perrett	2015	51	173	29	23-36
13	McQuaid	2015	42	50	84	74-94
15	Prymula	2014	55	182	30	24-37
Total			454	1651	**27**	**25 – 32**
**Test of Homogeneity: P < 0.05 Significant**						

Table S5. Frequency of local pain as an adverse effect of the 4CMenB vaccine in clinical trials

Study Number	Author	Year	Number of cases	Total number	Frequency (%)	95% CI
2	Beran	2021	87	94	92,6	87 – 98
3 (Fase 1)	Vesikari	2021	213	227	94	91 - 97
3 (Fase 2)	Vesikari	2021	115	126	91	86 - 96
5	Nolon	2019	250	254	98	97 - 99
6	Macias	2018	159	228	70	64 - 76
7	Chiu	2018	75	148	51	43 - 59
11 (Lote 1)	Perrett	2015	159	169	94	91 - 98
11 (Lote 2)	Perrett	2015	167	173	97	94 - 99
13	McQuaid	2015	23	50	46	32 - 60
15	Prymula	2014	63	182	35	28 - 42
19	Gossger	2012	2863	3330	86	85 - 87
Total			4174	4981	**84**	**83 - 85**
**Test of Homogeneity: P < 0.05 Significant**						

Table S6. Frequency of arthralgia as an adverse effect of the 4CMenB vaccine in clinical trials.

Study Number	Author	Year	Number of cases	Total number	Frequency (%)	95% CI
2	Beran	2021	56	94	59,6	50 – 69
3 (Fase 1)	Vesikari	2021	126	227	56	49 - 62
3 (Fase 2)	Vesikari	2021	64	126	51	42 - 60
5	Nolon	2019	140	254	55	49 - 61
11 (Lote 1)	Perrett	2015	60	169	36	28 - 43
11 (Lote 2)	Perrett	2015	61	173	35	28 - 42
19	Gossger	2012	1703	3330	51	49 - 53
Total			2210	4373	**51**	**49 - 52**
**Test of Homogeneity: P < 0.05 Significant**						

Table S7. Frequency of fatigue as an adverse effect of the 4CMenB vaccine in clinical trials

Study Number	Author	Year	Number of cases	Total number	Frequency (%)	95% CI
2	Beran	2021	39	94	41,9	32 – 51
3 (Fase 1)	Vesikari	2021	103	227	45	39 - 52
3 (Fase 2)	Vesikari	2021	61	126	48	39 - 52
5	Nolon	2019	125	254	49	43 - 55
11 (Lote 1)	Perrett	2015	54	169	32	25 - 39
11 (Lote 2)	Perrett	2015	63	173	19	13 - 25
13	McQuaid	2015	5	50	10	2 - 18
19	Gossger	2012	1412	3330	42	41 - 44
Total			1862	4423	**42**	**41 – 44**
**Test of Homogeneity: P < 0.05 Significant**						

Table S8. Frequency of nausea as an adverse effect of the 4CMenB vaccine in clinical trials

Study Number	Author	Year	Number of cases	Total number	Frequency (%)	95% CI
2	Beran	2021	1	94	1,1	0,2 – 5,8
3 (Fase 1)	Vesikari	2021	5	227	2	0,2 - 4
3 (Fase 2)	Vesikari	2021	6	126	5	1 - 8
4	O'Connor	2020	55	92	59,6	48,6 - 69,8
5	Nolon	2019	9	254	4	1 - 6
6	Macias	2018	54	228	24	18 - 29
7	Chiu	2018	71	148	48	40 - 56
11 (Lote 1)	Perrett	2015	5	169	3	0,1 - 6
11 (Lote 2)	Perrett	2015	4	173	2	0,1 - 5
13	McQuaid	2015	5	50	10	2 - 18
15	Prymula	2014	99	182	54	47 - 62
19	Gossger	2012	123	3329	4	3 - 5
Total			382	5072	**8**	**7 – 8,5**
**Test of Homogeneity: P < 0.05 Significant**						

Table S9. Frequency of headache as an adverse effect of the 4CMenB vaccine in clinical trials

Study Number	Author	Year	Number of cases	Total number	Frequency (%)	95% CI
2	Beran	2021	17	94	18,5	10 – 26
3 (Fase 1)	Vesikari	2021	25	227	11	11 - 25
3 (Fase 2)	Vesikari	2021	15	126	12	8 - 18
5	Nolon	2019	63	254	25	19 - 30
11 (Lote 1)	Perrett	2015	20	169	12	7 - 17
11 (Lote 2)	Perrett	2015	29	173	17	11 - 22
13	McQuaid	2015	10	50	20	9 - 31
Total			179	1093	**16**	**14 - 19**
**Test of Homogeneity: P > 0.05 Non-significant**						

Table S10. Frequency of myalgia as an adverse effect of the 4CMenB vaccine in clinical trials

Study number	Author	Year	Number of cases	Total number	Frequency (%)	95% CI
2	Beran	2021	38	94	40,4	30 – 50
3 (Fase 1)	Vesikari	2021	62	227	27	22 - 33
3 (Fase 2)	Vesikari	2021	22	126	17	11 - 24
5	Nolon	2019	98	254	39	32 - 44
11 (Lote 1)	Perrett	2015	90	169	54	46 - 61
11 (Lote 2)	Perrett	2015	102	173	59	52 - 66
Total			412	1043	**40**	**37 - 42**
**Test of Homogeneity: P < 0.05 Significant**						

Table S11. Frequency of fever as an adverse effect of the 4CMenB vaccine in clinical trials

Study Number	Author	Year	Number of cases	Total number	Frequency (%)	95% CI
2	Beran	2021	17	94	18,5	10 – 26
3 (Fase 1)	Vesikari	2021	42	227	19	13 - 24
3 (Fase 2)	Vesikari	2021	24	126	19	12 - 26
5	Nolon	2019	51	254	20	15 - 25
11 (Lote 1)	Perrett	2015	31	169	18	13 - 24
11 (Lote 2)	Perrett	2015	33	173	35	28 - 42
Total			198	1043	**19**	**17 - 21**
**Test of Homogeneity: P > 0.05 Non-significant**						

Table S12. Frequency of chill as an adverse effect of the 4CMenB vaccine in clinical trials

Study Number	Author	Year	Number of cases	Total number	Frequency (%)	95% CI
3 (Fase 1)	Vesikari	2021	45	227	20	15 – 25
3 (Fase 2)	Vesikari	2021	27	126	21	14 - 29
Total			72	353	**20**	**16 - 25**
**Test of Homogeneity: P > 0.05 Non-significant**						

Table S13. Frequency of loss of appetite as an adverse effect of the 4CMenB vaccine in clinical trials

Study Number	Author	Year	Number of cases	Total number	Frequency (%)	95% CI
3 (Fase 1)	Vesikari	2021	41	227	18	13 – 23
3 (Fase 2)	Vesikari	2021	20	126	16	9 - 22
6	Macias	2018	47	228	21	15 - 26
7	Chiu	2018	92	148	62	55 - 70
13	McQuaid	2015	17	50	34	21 - 47
15	Prymula	2014	42	182	54	47 - 62
Total			259	961	**27**	**24 - 30**
**Test of Homogeneity: P < 0.05 Significant**						

Table S14. Frequency of sleep disturbance as an adverse effect of the 4CMenB vaccine in clinical trials

Study Number	Author	Year	Number of cases	Total number	Frequency (%)	95% CI
6	Macias Parra	2018	78	228	34	28 – 40
7	Chiu	2018	79	148	53	45 - 61
13	McQuaid	2015	18	50	36	23 - 49
15	Prymula	2014	66	182	36	29 - 43
Total			241	608	**40**	**36 - 44**
**Test of Homogeneity: P > 0.05 Non Significant**						

Table S15. Frequency of persistent crying as an adverse effect of the 4CMenB vaccine in clinical trials

Study Number	Author	Year	Number of cases	Total number	Frequency (%)	95% CI
6	Macias Parra	2018	132	228	58	51 – 64
7	Chiu	2018	96	148	65	57 - 73
15	Prymula	2014	52	182	29	22 - 35
**Total**			**280**	**558**	**50**	**46 – 54**
**Test of Homogeneity: P < 0.05 Significant**						

Table S16. Frequency of vomiting as an adverse effect of the 4CMenB vaccine in clinical trials

Study Number	Author	Year	Number of cases	Total number	Frequency (%)	95% CI
6	Macias Parra	2018	28	228	12	8 – 17
7	Chiu	2018	21	148	14	9 - 20
13	McQuaid	2015	5	50	10	2 - 18
15	Prymula	2014	13	182	7	3 - 11
Total			67	608	**11**	**9 - 14**
**Test of Homogeneity: P > 0.05 Non-significant**						

Table S17. Frequency of diarrhea as an adverse effect of the 4CMenB vaccine in clinical trials

Study Number	Author	Year	Number of cases	Total number	Frequency (%)	95% CI
6	Macias Parra	2018	41	228	18	13 – 23
7	Chiu	2018	28	148	19	13 - 25
13	McQuaid	2015	4	50	8	0,1 - 16
15	Prymula	2014	31	182	17	12 - 22
Total			104	608	**17**	**14 - 20**
**Test of Homogeneity: P > 0.05 Non-significant**						

Table S18. Frequency of irritability as an adverse effect of the 4CMenB vaccine in clinical trials

Study Number	Author	Year	Number of cases	Total number	Frequency (%)	95% CI
6	Macias Parra	2018	121	228	53	47 – 60
7	Chiu	2018	111	148	75	68-82
13	McQuaid	2015	24	50	48	34-62
15	Prymula	2014	70	82	38	31-46
Total			326	508	**64**	**60 - 68**
**Test of Homogeneity: P < 0.05 Significant**						

Table S19. Frequency of skin rash as an adverse effect of the 4CMenB vaccine in clinical trials

Study Number	Author	Year	Number of cases	Total number	Frequency (%)	95% CI
6	Macias Parra	2018	28	228	12	8 – 17
7	Chiu	2018	18	148	12	7 - 17
11 (Lote 1)	Perrett	2015	7	169	4	1 - 7
11 (Lote 2)	Perrett	2015	7	148	4	1 - 7
13	McQuaid	2015	2	50	4	1 - 9
15	Prymula	2014	3	182	2	0,1 - 3
Total			65	925	**7**	**5 - 9**
**Test of Homogeneity: P < 0.05 Significant**						
